# Termination of wanted pregnancy and suicidal ideation in hyperemesis gravidarum: A mixed methods study

**DOI:** 10.1177/1753495X211040926

**Published:** 2021-10-19

**Authors:** Melanie Nana, Florence Tydeman, Georgie Bevan, Harriet Boulding, Kimberley Kavanagh, Caitlin Dean, Catherine Williamson

**Affiliations:** 1 Department of Obstetric Medicine, 8945 Guy's and St Thomas’ NHS Foundation Trust, UK; 2 Department of Mathematics and Statistics, 3527 University of Strathclyde, UK; 3 4922 British Broadcasting Corporation; BBC East, UK; 4 The Policy Institute, 388593 King's College London, UK; 5 Department of Mathematics and Statistics, 3527 University of Strathclyde, UK; 6 8945 Pregnancy Sickness Support, UK; 7 School of Life Course Sciences, King’s College London, UK

**Keywords:** Hyperemesis gravidarum, termination of pregnancy, suicidal ideation, treatment experience

## Abstract

**Background:**

Difficulty accessing medication and poor patient experience have been implicated as risk factors for termination of pregnancy and suicidal ideation in women with hyperemesis gravidarum. We aimed to gain further insight into these factors in order to further inform and improve patient care.

**Methods:**

We performed a sub-analysis on quantitative data generated through a UK-wide survey of 5071 participants. A qualitative analysis of free text comments was performed using an inductive thematic approach.

**Results:**

41.2% % of women taking prescribed medications had to actively request them. ‘Extremely poor’ or ‘poor’ experiences were described in 39.4% and 30.0% of participants in primary and secondary care respectively. Protective factors for termination of pregnancy and suicidal ideation include holistic support from family, friends and healthcare providers.

**Conclusion:**

Optimal care in hyperemesis gravidarum should incorporate timely access to pharmacotherapy, assessment of mental health, consideration of referral to specialist services and care being delivered in a compassionate manner.

## Introduction

Hyperemesis gravidarum (HG) is defined as persistent and excessive vomiting starting before the end of the 22nd week of gestation.^1^ It was reported as affecting 0.3–3.6% of pregnancies in a meta-analysis using data from 79 studies.^2^

Physical complications include weight loss, electrolyte disturbance, increased susceptibility to thrombosis and thiamine deficiency. Adverse impacts on mental health include increased anxiety, depression, postnatal depression and post-traumatic stress disorder.^3–5^ We recently reported termination of pregnancy (TOP) rates and suicidal ideation rates in 5071 women with a self-reported diagnosis of HG using an anonymous online survey. 4.9% of women surveyed reported having previously terminated a wanted pregnancy as a consequence of HG with a further 52.1% considering it. 25.5% and 6.6% of women reported occasional and regular suicidal ideation respectively. Both TOP and suicidal ideation were associated with increased severity of sickness, poor functional status and poor perception of quality of care received in both primary and secondary care. Women reporting ‘extremely poor’ quality care were less likely to have been offered medication to treat their symptoms compared with those reporting excellent care (odds ratio (OR), 23.99; 95% confidence intervals (CI), 17.79–32.74; OR, 7.39; 95% CI, 5.56–9.76).^6^

We aimed to gain further insight into contributory factors for both TOP and suicidal ideation, which may be able to inform and improve patient care by performing a sub-analysis of the original data and undertaking a qualitative analysis.

## Methods

This sub-analysis utilised data from a 14-item anonymous online survey (Supplementary Information 1). The survey was piloted by a patient advisory panel, with feedback incorporated. The form was circulated to members of the Pregnancy Sickness Support charity via email and posted on support forums/social media platforms.

Inclusion criteria included all women self-reporting a current or previous diagnosis of HG or severe sickness in pregnancy, and UK residency. Participants needed to be able to read and write in English or have access to a translator and required access to the internet. There were no specific exclusion criteria.

### Quantitative analysis

Analysis was performed as previously described.^6^ Chi-squared or fisher's exact test were used to evaluate relationships between outcomes. Odds ratios with 95% CI were calculated for binary outcomes using univariate logistic regression models. Bonferroni adjustment was applied: the significance level, α = 0.05, was divided by the total number of hypothesis tests carried out (n = 48) therefore adjusting the significance level to α = 0.001. These data included missing responses and ‘Don’t Know’/‘Not Applicable’ responses which were handled using pairwise deletion (Supplementary Information 2). New variables were derived from some survey questions, by combining responses, to create binary outcomes and present the data more succinctly (Supplementary Information 3).

### Qualitative analysis

Comments captured in free text boxes were analysed using an inductive thematic approach using qualitative data processing with NVivo for Mac (v12). The data were coded, categorised and reviewed for emergent themes. Initial analysis was undertaken by Melanie Nana; then refined through discussion with Harriet Boulding and Catherine Williamson.

### Patient and public involvement

Caitlin Dean is a patient representative and her position ensured participant's voices were central to the design, piloting, data collection, analysis and interpretation. The online HG patient community has been utilised to ensure broad public dissemination of the results. The intentions of the survey were clearly outlined and as such participant consent was assumed by voluntary involvement.

### Ethical approval

The King's College London Research and Ethics Committee advised that ethical approval was not required on the basis that all data was anonymous with no means of accessing identifiable information or linking any data.

## Quantitative analysis

### Demographics

A total of 5071 participants completed the survey from 14 regions across the UK (geographical distribution described in Supplementary Information 4). At the time of completing the survey, 18.6% (940/5057) were experiencing symptoms; 28.3% (1430/5057) had experienced symptoms in the past year. Of those that responded to the question regarding the number of affected pregnancies, 43.7% (2215/5064) reported severe sickness in one pregnancy, the remainder in multiple pregnancies.

### Management

In total, 85.7% (4172/4863) of participants reported taking prescribed medication, however, 41.2% (2004/4863) of those had to actively request medication (i.e. the consideration of starting pharmacotherapy was initiated by the patient as opposed to the healthcare professional). 48.7% (2370/4863) of people were offered medication, and 91.5% (2004/2370) of those offered, took the medication. Rehydration therapy was given to 69.0% (3483/5054). Those who took medication or received rehydration therapy were more likely to self-report a diagnosis of HG (OR = 12.16, 95% CI 10.10–14.69 and OR = 15.06, 95% CI 12.94–17.55, respectively). There was no association between geographical location and women who were offered medication or received rehydration therapy (*p* > 0.001 and *p* > 0.001).

#### Treatment experience

In total, 15.3% (732/4796) and 24.1% (1158/4796) perceived their primary care experience as ‘extremely poor’ and ‘poor’, respectively. 9.9% (440/4424) and 20.1% (887/4424) perceived their secondary care experience as ‘extremely poor’ or ‘poor’, respectively.

Those who reported ‘extremely poor’ experience in primary and secondary care were more likely to have not taken medication (for any reason, not specifically because medication had not been prescribed) and not receive rehydration treatment, compared to those reporting ‘excellent’ care: primary care (OR = 8.14, 95% CI 5.00–14.13 and OR = 1.90, 95% CI 1.47–2.46) and secondary care (OR = 15.14, 95% CI 9.06–26.99 and OR = 8.00, 95% CI 5.87–11.03) (see Table 1 for full univariate regression results). There was no association between treatment experience in primary or secondary care and geographical location (*p* > 0.001 and *p* > 0.001)).

**Table 1.** Women that received medication or rehydration treatment had a better perception of primary and secondary care experience.

	No medication taken compared with medication taken		No rehydration treatment compared with rehydration treatment given	
	Odds Ratio (95% CI) and chi-squared *p*-value			
Treatment experience primary care				
Excellent	1	*p* < 0.001	1	*p* < 0.001
Good	2.46 (1.48–4.34)		1.57 (1.23–2.01)	
Satisfactory	4.48 (2.76–7.74)		2.05 (1.62–2.61)	
Poor	6.59 (4.09–11.34)		1.83 (1.44–2.33)	
Extremely poor	8.14 (5.00–14.13)		1.90 (1.47–2.46)	
Treatment experience secondary care				
Excellent	1	*p* < 0.001	1	*p* < 0.001
Good	3.62 (2.16–6.45)		1.95 (1.46–2.65)	
Satisfactory	6.78 (4.13–11.92)		4.03 (3.05–5.40)	
Poor	11.09 (6.78–19.45)		6.10 (4.60–8.18)	
Extremely poor	15.14 (9.06–26.99)		8.00 (5.87–11.03)	

Note: Odds ratios (OR) with 95% confidence intervals (CI) from univariate logistic regression model.

### Daily life

Regarding functional status, 67.8% (3432/5016) were ‘bedridden most of the time needing daily extra support’. Those reporting poor functional status were more likely to self-report a diagnosis of HG compared to those who could function most of the time (OR = 33.55, 95% CI 22.40–52.10 and OR = 5.05, 95% CI 4.38–5.81): defined as ‘bedridden most of the time and needing daily support’ and ‘able to function some of the time and needed extra support’, in comparison to ‘able to function most of the time’.

## Qualitative analysis

5500 free text comments were stratified into 65 codes then grouped into 19 categories. Five overarching themes emerged (Table 2).

**Table 2.** Emergent themes from qualitative analysis with representative quotations.

Categories	Comment frequency	Representative quotations
**Social outcomes**		
Effect on daily life	328	‘Being unable to do basic things like shower or even sit up, left me in absolute despair’ ‘Before I found medication that worked, I was bedridden, with a bed sore on my hip and could not move to ring the doctor’
Ability to care for family	179	‘The smell of my children made me sick, I let them down as I could not look after them’ ‘It almost ruined my relationship and bond with my first child as I barely saw her as I spent so much time in hospital’
Family and friends	482	‘My family thought I was being lazy and just needed to get out of bed’ ‘Even my own family accused me of hurting my baby because I was on constant medication’
Financial burden	70	‘Work did not understand and eventually my hours were dropped and I began to get into debt’ ‘Being self-employed I lost all of my income’
Work	369	‘Now my baby is 9 months old I am making arrangements to go back to work, but they are trying to push me out. I feel that this is due to the length of time I had off sick. I have now had to move to another lower paid role’
**Overall management**		
Treatment satisfaction	1117	‘Hardest thing after 11 admissions was midwife saying “You ladies with HG take up so much of my time” she was rude and made me feel so uneasy and low I cried a whole 12 h, but I was too scared to complain’ ‘I was referred to a “special team”, I thought it was to help with the HG, but I had been referred on mental grounds’
Management	331	‘Measuring for ketones would have indicated that I was not unwell even when I was extremely dehydrated. Thankfully I was generally assessed and given appropriate treatment’
Support and awareness	654	‘There is not enough awareness of HG across the medical professionals or general public’ ‘I just wish people understood how debilitating and humiliating it is’
**Physical and mental outcomes**		
Physical health	1158	‘My partner recalls being able to see my bones protruding through my skin due to the weight loss’
Multiple pregnancies affected	237	‘I suffered in all three pregnancies, each getting worse’
Mental health	308	‘I suffered extreme anxiety and was told the waiting list for cognitive behavioural therapy was 5 months although I never received an appointment’
Suicidal ideation	65	‘After trying for a baby for years and resorting to in vitro fertilization I struggled with the feelings that I wanted to die’ ‘I sobbed when I awoke in the mornings because I realised, I was still alive’
**Pregnancy outcomes**		
Concern regarding pregnancy outcome	135	‘I took medication for sickness and my baby was born visually impaired. We do not know whether it was coincidental or not, but I will always blame myself’
Termination of pregnancy	223	‘At the point of termination, it was either an abortion or suicide’ ‘I had no other option if I was going to be able to look after my existing child’
**Long-term effects**		
Physical health	26	‘I am still having dental treatment as my previously healthy teeth started to decay’
Mental health	92	‘Led to long-term depression and attempted suicide’ ‘HG had a long-term impact on my relationship with food, I never regained a healthy weight’
Effect on desire for future pregnancy	359	‘I have been sterilised because the thought of another pregnancy with HG terrifies me’ ‘A further pregnancy would kill me and risk my marriage; I am terrified of having sex despite having the coil inserted’ ‘I cried for days when I found out I was pregnant again; I really had no idea how we could get through it’ ‘I always wanted a big family but if I were to become pregnant again, I would terminate immediately- something I would never have considered prior to suffering with HG’

HG had marked and deleterious effects on affected women's ability to function on a daily basis and to look after their families. Inability to work increased feelings of isolation and financial strain. One woman described making the decision to terminate ‘to avoid losing my new job and home for my first child, which I had rented after 6-months of homelessness’. Another commented that ‘I became homeless because I couldn’t work, I was sick and wished I’d die, I was hopeless, sick and scared’.

Women expressed difficulty in accessing treatment. One commented that ‘any requests for help were met with “you will have to visit your GP” which was impossible as I couldn’t leave my bed’. Other typical comments included ‘I was made to feel stupid when I reported the extent of my sickness and that I should really have been able to cope’. Many descriptions of attitudes of health care professionals were negative and revealed a lack of knowledge surrounding HG. Unanswered questions and lack of clarity regarding the safety of medication increased anxiety about the health of the unborn child. One woman was told ‘It is better to be incapacitated and unable to look after your pre-existing child than to risk your unborn baby’ when she requested an antiemetic.

Lack of support from the public and family members exacerbated feelings of despair. Two key influences emerged as risk factors for termination 1) being unable to look after existing children 2) feeling this was the only option to prevent suicidal ideation. Women described their experience of termination as ‘distressing’ and ‘harrowing’; one woman recalls that ‘as I was signing the form, I looked to the doctor for some comfort that my unborn baby would not feel pain and her response was “well it does have a nervous system”’. Others described their feelings years later ‘I don’t think I will ever get over it. I cry as I write this. I am grateful for the child that I have but will never forget the one that I lost, but I had no other option at the time’.

In terms of suicidal ideation one woman described becoming ‘so ill that I considered termination, when I couldn’t bring myself to do that, I contemplated taking my own life’. Another commented that her ‘mental state became so bad that I attempted suicide twice’. Support from spouse, family and the health care community emerged as protective factors: ‘I was in a dark place and wanted to die, I dread to imagine what would have happened without the support of my family and midwife’. Women described how death felt preferable to constant nausea and vomiting, with 19 women stating that they ‘hoped to not wake up each morning’. There were 74 comments relating to anxiety regarding future pregnancy, including women describing being ‘fearful’, ‘petrified’ and ‘terrified’. There were 99 references regarding being unable to cope with a further pregnancy including ‘I’d like more children, but don’t think my body or mind could take the strain’. A further 184 women described having made decisions to not undergo further pregnancy as a consequence of HG, one woman stating that ‘I know that I am never going to suffer HG again after taking the easy decision to be sterilised, simply because I could not survive another pregnancy’.

## Discussion

This sub-analysis describes characteristics of women self-reporting a diagnosis of HG across the UK. It further highlights challenges in accessing pharmacotherapy for the management of HG, which have been demonstrated to contribute towards increased rates of termination of a wanted pregnancy and suicidal ideation. Qualitative analysis revealed contributing influences for TOP and suicidal ideation.

In the short term almost 70% of women in this study were bedridden, reducing their ability to look after themselves, their family and continue employment. Several studies report rates of absence from work between 25.0%–46.5%, some describing job loss, resulting in financial burden.^4,7−9^

In one study 46.5% of women not able to work and therefore on sickness absence had not received pharmacological treatment beforehand.^8^ In our study, although 85.7% of women took prescribed medication, 41.2% had to actively request it. In a survey carried out by two UK charities, nearly half of women with HG who terminated their pregnancy had either not been offered, or had been declined medication.^10^ The complications of thalidomide use in the 1960s resulted in all medications in pregnancy being suspected of teratogenicity and thus a reluctance to prescribe is often based around concerns regarding impact on the fetus and lack of safety data.^7,11^ Reassuringly, a Cochrane review and other systematic reviews/meta-analyses have now reported on the safety and efficacy of many antiemetic drugs in pregnancy with no increased risk of teratogenesis or other adverse outcomes.^12,13^ A risk/benefit decision should be made between prescribing such medications and the risks of untreated HG. In addition, women describe guilt when taking antiemetics due to concerns about causing harm to their baby and are not supported in doing so by family members, resulting in poor compliance.

Our qualitative analysis identified 65 individual comments relating to suicidal ideation. Of these, 23 women believed there was little hope of accessing help and thus ‘no other way out’. Referral to a mental health team providing individualised emotional and psychological support improves outcomes and quality of life and therefore should be made where appropriate.^14^ This is particularly pertinent considering that maternal suicide currently represents the second most common cause of direct maternal death in the UK.^15^ Risk factors for suicide include social isolation and pregnancy complications, both frequently observed in women with HG.^16^

Rates of termination as high as 15.2% in women with HG have been previously described.^4,9,17^ Common reasons in one survey included inability to care for family and self (66.7%) and fear that they or their baby could die (51.2%)^17^ consistent with the results of this study. Women who terminate have been described as being three times more likely to state their healthcare professionals (HCPs) were uncaring compared to those who did not.^17^ Furthermore, in this cohort 223 make comments regarding termination, many describing themselves in situations in which ‘at the point of termination, it was either an abortion or suicide’.

We have demonstrated a clear association between perception of care received from HCPs and poor mental health outcomes/termination of pregnancy.^6^ A number of studies have highlighted women feeling stigmatised by HCPs, labelled as ‘problem patients’.^9,18^ An ongoing belief exists that severe sickness is a psychological disorder; with 7.8% of women in one study stating that they had been told their condition was psychological.^17^ Reported explanations range from describing HG as a conversion disorder, hysteria or a product of conditioning.^3^ More recent, higher-quality studies have rejected these hypotheses.^9,19^ In our cohort, 441 women reported that they experienced nausea and/or vomiting 20 times/day or more but did not report a diagnosis of HG, questioning whether women with significant disease are being given an appropriate diagnosis and therefore appropriate care.

Further work is required to explore the experience of those currently underrepresented such as those in certain geographical areas. Prospective work would benefit from further data on socioeconomic and previous mental health factors to allow better account for confounders.

### Strengths and limitations

Our study represents the largest to date exploring the experience of women with severe sickness in pregnancy. Strengths include a wide geographical spread and its mixed methods nature. As described previously limitations include data being collected from women who self-reported a diagnosis of HG or severe sickness in pregnancy without confirmation of diagnosis. Due to limited space the questionnaire asked about nausea and vomiting together, and a future study would aim to ask about each of these features of HG separately. The online nature of the survey meant certain population groups are likely to be underrepresented. For example, those from socioeconomic groups without access to the internet or those unable to read or speak English. In addition, there is a risk of selection bias overrepresenting, for example, those with more severe disease.

## Conclusion

HG has been demonstrated to increase rates of TOP and suicidal ideation. While we acknowledge the pressures experienced in both primary and secondary care, HCPs should adopt a holistic and compassionate approach. This should incorporate prescription of pharmacotherapy where indicated, mental health assessment, consideration of referral to specialist services where required while taking into account the social consequence of the disease on the patient and their family.

## Supplemental Material

sj-docx-1-obm-10.1177_1753495X211040926 - Supplemental material for Termination of wanted pregnancy and suicidal ideation in hyperemesis gravidarum: A mixed methods study Click here for additional data file. Supplemental material, sj-docx-1-obm-10.1177_1753495X211040926 for Termination of wanted pregnancy and suicidal ideation in hyperemesis gravidarum: A mixed methods study by Melanie Nana, Florence Tydeman, Georgie Bevan, Harriet Boulding, Kimberley Kavanagh, Caitlin Dean and Catherine Williamson in Obstetric Medicine

sj-docx-2-obm-10.1177_1753495X211040926 - Supplemental material for Termination of wanted pregnancy and suicidal ideation in hyperemesis gravidarum: A mixed methods study Click here for additional data file. Supplemental material, sj-docx-2-obm-10.1177_1753495X211040926 for Termination of wanted pregnancy and suicidal ideation in hyperemesis gravidarum: A mixed methods study by Melanie Nana, Florence Tydeman, Georgie Bevan, Harriet Boulding, Kimberley Kavanagh, Caitlin Dean and Catherine Williamson in Obstetric Medicine

sj-docx-3-obm-10.1177_1753495X211040926 - Supplemental material for Termination of wanted pregnancy and suicidal ideation in hyperemesis gravidarum: A mixed methods study Click here for additional data file. Supplemental material, sj-docx-3-obm-10.1177_1753495X211040926 for Termination of wanted pregnancy and suicidal ideation in hyperemesis gravidarum: A mixed methods study by Melanie Nana, Florence Tydeman, Georgie Bevan, Harriet Boulding, Kimberley Kavanagh, Caitlin Dean and Catherine Williamson in Obstetric Medicine

sj-docx-4-obm-10.1177_1753495X211040926 - Supplemental material for Termination of wanted pregnancy and suicidal ideation in hyperemesis gravidarum: A mixed methods study Click here for additional data file. Supplemental material, sj-docx-4-obm-10.1177_1753495X211040926 for Termination of wanted pregnancy and suicidal ideation in hyperemesis gravidarum: A mixed methods study by Melanie Nana, Florence Tydeman, Georgie Bevan, Harriet Boulding, Kimberley Kavanagh, Caitlin Dean and Catherine Williamson in Obstetric Medicine
