# Supplementary material for: Termination of wanted pregnancy and suicidal ideation in hyperemesis gravidarum: A mixed methods study
Source: Obstet Med. 2021 Oct 19;15(3):180–4. doi: 10.1177/1753495X211040926 (PMC9574451; doi:10.1177/1753495X211040926)
Supplement: sj-docx-1-obm-10.1177_1753495X211040926 - Supplemental material for Termination of wanted pregnancy and suicidal ideation in hyperemesis gravidarum: A mixed methods study [file sj-docx-1-obm-10.1177_1753495X211040926.docx]

**Supplementary Information 1: Details of the survey**

| **Information collected** | **Question Format** | **Multiple choice answers available** |
| --- | --- | --- |
| 1. Where do you live in the UK? | MCQs | England- North West; England- East; England- East Midlands; England- London; England- North East; England- South; England- South East; England-South West; England- West, England- West Midlands; England- Yorkshire/Hull; Northern Ireland; Scotland; Wales |
| 2. When did you last experience severe sickness in pregnancy? | MCQs | I am experiencing it now; in the last year; in the last 3 years; more than 4 years ago |
| 3. At its worst, what was your level of sickness in pregnancy. | MCQs | Diagnosed with Hyperemesis Gravidarum – HG; nausea and/or vomiting at least 20 times a day; nausea and/or vomiting at least 10 times a day; nausea and/or vomiting at least 5 times a day; constant nausea and occasional/no vomiting; occasional nausea and vomiting |
| 4. How many of your pregnancies resulted in extreme sickness? | MCQs | 1 pregnancy; 2 pregnancies; 3 or more pregnancies |
| 5. When you were experiencing extreme sickness, to what extent did it affect your daily life? | MCQs | I was bedridden most of the time and needed daily support; I was able to function some of the time and needed extra support; I was able to function most of the time; don’t know |
| 6. To what extent did you consider terminating your pregnancy because you felt so sick? | MCQs | I terminated my pregnancy; I seriously considered it; I considered it only briefly; I never considered it; Don't know |
| 7. Because of extreme sickness and the impact on your mental health, did you ever consider suicide? | MCQs | Yes, I had regular suicidal thoughts; Yes, I had suicidal thoughts on occasion; Yes, I once had suicidal thoughts; No, I never had suicidal thoughts; Not Applicable |
| 8. To what extent did extreme sickness in pregnancy affect your longer-term mental health? | MCQs | It had an extremely negative impact; it has some negative impact; it has no impact; it has some positive impact; it had an extremely positive impact; don’t know |
| 9. To what extent did extreme sickness in pregnancy affect your longer-term physical health? | MCQs | It had an extremely negative impact; it has some negative impact; it has no impact; it has some positive impact; it had an extremely positive impact; don’t know |
| 10. Did you take prescribed medication to help reduce your extreme sickness? | MCQs | Yes, I was quickly offered prescribed medication and took it; yes, I took prescribed medication but had to request it; no, I did not take prescribed medication despite requesting it; no, I was offered prescribed medication but chose not to take it; no, I was not offered prescribed medication and did not take it; don’t know |
| 11. Because of extreme sickness, were you given rehydration treatment during your pregnancy? | MCQs | Yes, I stayed overnight in hospital for treatment at least once; yes, I was given treatment as a hospital outpatients/clinic day patient at least once; Yes, I was treated at home under medical supervision; No, I did not receive any rehydration; don’t know |
| 12. How would you describe the overall advice/treatment you received from your GP surgery for extreme sickness in pregnancy? | MCQs | Excellent; good; satisfactory; poor; extremely poor; not applicable |
| 13. How would you describe the overall advice/treatment you received in hospital/clinic for extreme sickness in pregnancy? | MCQs | Excellent; good; satisfactory; poor; extremely poor; not applicable |
| 14. If you would like to tell us more about your experience of extreme sickness in pregnancy, use the comments box below. | CB | NA |

Abbreviations: MCQs = Multiple choice questions; CB = Comment box ; NA = not applicable
