# Supplementary material for: Termination of wanted pregnancy and suicidal ideation in hyperemesis gravidarum: A mixed methods study
Source: Obstet Med. 2021 Oct 19;15(3):180–4. doi: 10.1177/1753495X211040926 (PMC9574451; doi:10.1177/1753495X211040926)
Supplement: sj-docx-2-obm-10.1177_1753495X211040926 - Supplemental material for Termination of wanted pregnancy and suicidal ideation in hyperemesis gravidarum: A mixed methods study [file sj-docx-2-obm-10.1177_1753495X211040926.docx]

Multiple responses

Participants could select multiple answers per question which allowed for a greater number of responses than total number of total participants. Multiple responses from the survey were handled in one of two ways:

1. For questions where multiple responses would introduce bias the participant was removed.
2. For questions presented in a scaled format the participant’s ‘most extreme’ answer was chosen.

**Supplementary Information 2: Questions with multiple responses. The table below explains how ‘multiple responses’ have been handled for each question.**

| **Question** | **No. non respondents** | **No. Don't Know (DK) or Not Applicable (NA)** | **No. Repeated Responses** | **No. Responses per Question** | **Handling of Multiple Responses** | **(%) of Total Responses Missing** |
| --- | --- | --- | --- | --- | --- | --- |
| Location | 42 | - | - | 5029 | - | 0.83% |
| Last Experience | 14 | - | 371 | 5057 | Most extreme answer selected | 0.28% |
| Level of Sickness | 55 | - | 703 | 5016 | Most extreme answer selected | 1.08% |
| Number of Pregnancies | 7 | - | 7 | 5064 | Most extreme answer selected | 0.14% |
| Daily Life | 4 | 4 - DK | 111 | 5063 | Most extreme answer selected | 0.16% |
| Medication | 54 | 16 - DK | 138 | 4863 | Multiple responses removed | 4.10% |
| Rehydration Treatment | 7 | 10 - DK | 181 | 5054 | Most extreme answer selected | 0.34% |
| Treatment Experience GP | 20 | 136 - NA | 119 | 4796 | Multiple responses removed | 5.42% |
| Treatment Experience Hospital | 13 | 528 - NA | 106 | 4424 | Multiple responses removed | 12.76% |
| Termination | 10 | 67 - DK | 83 | 4994 | Most extreme answer selected | 1.52% |
| Suicidal Ideation | 7 | 100 - NA | 30 | 4964 | Most extreme answer selected | 2.11% |
| Long Term Mental Health | 14 | 242 - DK | 56 | 4759 | Multiple responses removed | 6.15% |
| Long Term Physical Health | 26 | 365 - DK | 47 | 4633 | Multiple responses removed | 8.64% |
