# Supplementary material for: Termination of wanted pregnancy and suicidal ideation in hyperemesis gravidarum: A mixed methods study
Source: Obstet Med. 2021 Oct 19;15(3):180–4. doi: 10.1177/1753495X211040926 (PMC9574451; doi:10.1177/1753495X211040926)
Supplement: sj-docx-3-obm-10.1177_1753495X211040926 - Supplemental material for Termination of wanted pregnancy and suicidal ideation in hyperemesis gravidarum: A mixed methods study [file sj-docx-3-obm-10.1177_1753495X211040926.docx]

**Supplementary Information 3: Definition of combined response variables. The table below explains how survey variables were combined to create new/ more manageable versions of the survey questions.**

| **Survey Variable** | **Category of Response** | **Combined Categories from Original Survey Question** |
| --- | --- | --- |
| Self-reported diagnosis of HG | Yes/ No | Yes = “Diagnosed with Hyperemesis Gravidarum – HG”  No = All other responses |
| Medication Offered | Yes/ No | Yes = “Yes, I was quickly offered prescribed medication and took it” & “no, I was offered prescribed medication but chose not to take it”  No = “yes, I took prescribed medication but had to request it” & “no, I did not take prescribed medication despite requesting it” & “no, I was not offered prescribed medication and did not take it” |
| Medication Taken | Yes/ No | Yes = “Yes, I was quickly offered prescribed medication and took it” & “Yes, I took prescribed medication but had to request it”  No = “no, I did not take prescribed medication despite requesting it” & “no, I was offered prescribed medication but chose not to take it” & “no, I was not offered prescribed medication and did not take it” |
| Rehydration Treatment | Yes/ No | Yes = “Yes, I stayed overnight in hospital for treatment at least once” & “; yes, I was given treatment as a hospital outpatients/clinic day patient at least once” & “Yes, I was treated at home under medical supervision”  No = “No, I did not receive any rehydration” |
| Suicidal Ideation | Regular/ Occasional/ Never | Regular = "Yes, I had regular suicidal thoughts"  Occasional = "Yes, I had suicidal thoughts on occasion” & “Yes, I once had suicidal thoughts"  Never = "No, I never had suicidal thoughts" |
| Termination | Confirmed/ Considered/ Never Considered | Confirmed = "I terminated my pregnancy"  Considered = "I seriously considered it" & "I considered it only briefly"  Never Considered = "I never considered it" |
