# Supplementary material for: Termination of wanted pregnancy and suicidal ideation in hyperemesis gravidarum: A mixed methods study
Source: Obstet Med. 2021 Oct 19;15(3):180–4. doi: 10.1177/1753495X211040926 (PMC9574451; doi:10.1177/1753495X211040926)
Supplement: sj-docx-4-obm-10.1177_1753495X211040926 - Supplemental material for Termination of wanted pregnancy and suicidal ideation in hyperemesis gravidarum: A mixed methods study [file sj-docx-4-obm-10.1177_1753495X211040926.docx]

**Supplementary Information 4: Table showing number of participants per location across the UK**
